# Aging Alters Hair Cell Physiological Properties in Mice With Late‐Onset Age‐Related Hearing Loss

**DOI:** 10.1111/acel.70679

**Published:** 2026-08-21

**Authors:** Piece Yen, Adam J. Carlton, Andrew O'Connor, Niovi Voulgari, Stuart L. Johnson, Jing‐Yi Jeng

**Affiliations:** ^1^ School of Biosciences University of Sheffield Sheffield UK; ^2^ Neuroscience Institute University of Sheffield Sheffield UK

**Keywords:** age‐related hearing loss, cochlear aging, cochlear hair cell, potassium current

## Abstract

Mammalian cochlear hair cells convert acoustic stimuli into electrical signals, which are relayed to the central auditory pathway via auditory neurons. Progressive loss of hair cells and their synapses is a characteristic of age‐related hearing loss (ARHL), but the underlying mechanisms remain poorly understood. In this study, we tested the hypothesis that hair cell physiological properties change with age in CBA/CaJ mice, a strain known for its slow ARHL progression. We found that hair cell size began to decrease from 10 months of age, well before detectable increase in hearing thresholds from auditory brainstem responses. This structural change was associated with a significant decrease in the size of the basolateral membrane current, in particular that carried by the large conductance calcium‐activated potassium (BK) channel currents (*I*_K,f_) in the apical inner hair cells (IHCs). However, the size of the mechanoelectrical transducer (MET) current measured in the IHCs remained unaffected at least up to 22 months of age. Our results provide clear evidence of an early change in the physiological properties of apical IHCs with age, which precedes measurable hearing loss, suggesting that these cellular deficits might define a distinct pathological route in the CBA/CaJ mice.

## Graphical abstract

Hidden hearing loss during aging is associated with peripheral auditory system decline. We discovered that aging sensory hair cells decrease in size. In addition, inner hair cells exhibit a reduced K^+^ current carried out by BK channels, accompanied by an elevated resting membrane potential, suggesting an age‐related functional impairment.

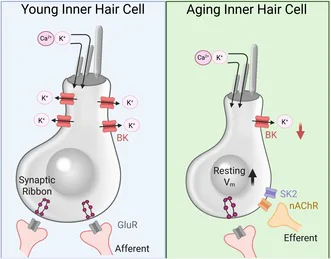

## 1 Introduction

ARHL, also known as presbycusis, is one of the most common age‐related disorders affecting over one third of adults over 60 years old (Cieza et al. 2021; World Report on Hearing 2021). Despite its high prevalence, the mechanisms underlying ARHL are still unclear due to its complexity. The progression of ARHL is highly variable in humans, starting between 30 and 70 years of age (Yamasoba et al. 2013). It involves various pathologies, which can differ among individuals, including the degeneration of spiral ganglion neurons (synaptopathy of SGNs) and the sensory hair cells within the cochlea (Gacek and Schuknecht 1969; Wu et al. 2018). Furthermore, ARHL is linked to risk factors, such as genetic predispositions, environmental insults, and lifestyle choices (Gioacchini et al. 2023; Tang et al. 2023; Wu et al. 2021). To study these mechanisms, various mouse models that represent different patterns of age‐related hearing loss have been used over decades. For example, C57BL/6 mice model early‐onset ARHL, while CBA mice model late‐onset ARHL. Hearing threshold measured from auditory brainstem responses (ABRs) can begin to elevate in C57BL/6 mice before 3‐months of age, in contrast to 18–20 months of age in CBA mice (Li and Borg 1991; Jeng, Ceriani, et al. 2020; Park et al. 2024). Although the factors contributing to the variability within and among these models are still unclear, they generally share similar underlying pathologies such as hair cell and auditory neuron loss (Li and Hultcrantz 1994; Spongr et al. 1997; Sha et al. 2008). Importantly, auditory neuron loss might not alter hearing thresholds, but it can lead to a reduction in auditory nerve responses, a condition called hidden hearing loss (HHL; C Kohrman et al. 2020).

There are two types of sensory cells in the mammalian cochlea: inner hair cells (IHCs) and outer hair cells (OHCs). OHCs provide acoustic amplification, while IHCs are the primary sound‐transducing sensors. Both cell types are depolarized when sound‐induced deflection of their stereociliary bundles causes the opening of the mechanically‐sensitive mechanoelectrical transducer (MET) channels located at the tips of the shorter stereociliary rows (Beurg et al. 2009). As MET channels open, an influx of cations depolarizes the cell, generating a receptor potential that triggers the release of synaptic vesicles onto SGN terminals. This receptor potential is a graded and sustained response, modulated by K^+^ currents that are crucial for regulating the resting membrane potential and preventing action potentials. Mature hair cells express characteristic K^+^ currents: *I*_K,n_ and *I*_K,f_. *I*_K,n_ is a negatively‐activating current carried out by KCNQ channels, and *I*_K,f_ is a fast K^+^ current mediated by the activation of large‐conductance Ca^2+^‐activated K^+^ channels (BK channels) (Kros et al. 1998; Marcotti and Kros 1999; Marcotti et al. 2003; Oliver et al. 2003). Across all cochlear regions, OHCs predominantly express *I*_K,n_, while IHCs express *I*_K,f_ (Rohmann et al. 2015; Wersinger et al. 2010; Johnson 2015). In an early‐onset ARHL mouse model, C57BL/6N, hair cells show a progressive reduction in the size of *I*_K,f_ and MET currents (Jeng, Johnson, et al. 2020; Jeng, Carlton, et al. 2021), suggesting that hair cells functionally degenerate with age. However, it is unclear if these functional changes occur across all ARHL mouse models.

In contrast to C57BL/6N mice, CBA mice develop a much later‐onset ARHL associated with hair cell loss, making them an ideal model to study slow ARHL (Ohlemiller et al. 2010; Sha et al. 2008; Spongr et al. 1997). It is well established in morphological investigations that IHCs in CBA mice begin to lose their presynaptic ribbons before hearing threshold changes are evident, a phenomenon known as hidden hearing loss (Sergeyenko et al. 2013; Liberman and Kujawa 2017; Parthasarathy and Kujawa 2018; Dörje et al. 2024). This suggests a possible pathological mechanism within IHCs that contributes to later hearing decline.

Here we test the hypothesis that hair cells undergo functional degeneration before the onset of ARHL. Using patch‐clamp electrophysiology and imaging, we investigated physiological properties of IHCs and OHCs from aging CBA/CaJ mice. We found that both types of hair cells are smaller with age and that the size of *I*_K,f_ started to decrease in IHCs much earlier than the onset of hearing threshold shift. Unlike previous observations in C57BL/6N mice, we found no evidence of stereociliary abnormalities from electron microscopy, and the size of MET current is maintained in IHCs with age. Similar to C57BL/6N mice (Zachary and Fuchs 2015; Jeng, Carlton, et al. 2021), aging IHCs in CBA/CaJ mice were also innervated by efferent neurons after the changes in the IHC current profile. These findings underscore that physiological change in hair cells is a key, early factor contributing to the pathogenesis associated with ARHL.

## 2 Results

### 2.1 CBA/CaJ Mice Show Mild Hearing Loss With Age

**FIGURE 1:**
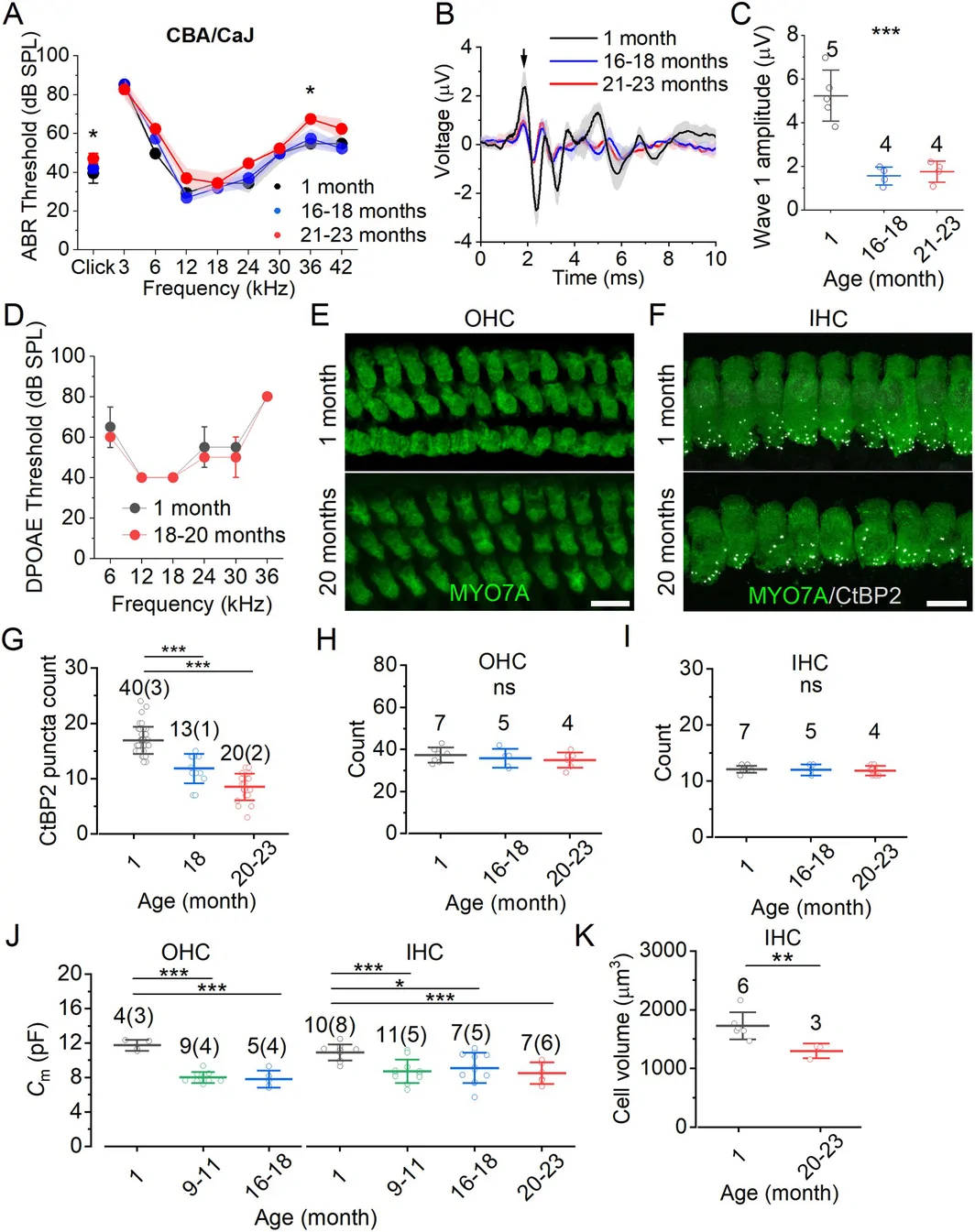
Hearing thresholds and hair cell morphology in aging CBA/CaJ mice. (A–C) ABR recordings for click and pure‐tone stimulation of 3–42 kHz recorded from CBA/CaJ mice at 1, 16–18 and 21–23 months. ABR thresholds from CBA/CaJ mice (A) are shown as median ± median absolute deviation (MAD), *p < 0.05, Kruskal–Wallis ANOVA. Average ABR waveform responses (B) recorded at 12 kHz and at 70 dB from CBA/CaJ mice are shown as mean ± SD. Average amplitude of wave 1 (arrow in B and E) is plotted as mean ± SD (C). Single‐animal recordings are plotted as open symbols. The number of CBA/CaJ animals in A–C is shown in C. (D) DPOAE thresholds measured from 1 and 18–20 months CBA/CaJ mice. Data are median ± MAD, *p* > 0.5, Kruskal–Wallis ANOVA. (E and F) Maximum intensity projections of confocal *z*‐stacks of OHCs (E) and IHCs (F) from CBA/CaJ mice at 1 month and 20 months. The cells were labeled with antibodies against a hair cell marker MYO7A (green) and presynaptic ribbon marker CtBP2 (gray). (G) Quantification of the number of CtBP2 puncta per IHC from anti‐CtBP2 confocal images of aging CBA/CaJ mice. (H and I) Quantification of the number of cells in aging CBA/CaJ mice. The number of cells was counted across a 100 μm region from the apical coil of mice at 1, 16–18 and 20–23 months, and the values from each mouse were plotted behind the mean ± SD values. One‐way ANOVA, ns, not significant. *J*, cell membrane capacitance (*C*_m_) of IHCs and OHCs in aging CBA/CaJ mice. Recordings from each cell are shown behind the mean ± SD values. The number of cells (animals) are indicated above each data set. ***p < 0.001, **p < 0.01, *p < 0.05, one‐way ANOVA followed by Tukey's post hoc test. (K) The volume of IHCs quantified from confocal images across a 100 μm region from the apical coil of CBA/CaJ mice at 1 and 20–23 months. *p* = 0.009, *t*‐test.

We first determined the hearing thresholds in aging CBA/CaJ mice by measuring ABRs. As expected, these mice show little change in ABR thresholds with age where the thresholds were elevated only when stimulated with a click or a pure tone at 36 kHz at 21–23 months (Figure 1A, *p* = 0.031 and 0.028, respectively).

To further assess the output from the cochlea, we analyzed ABR wave 1 generated by the auditory nerve that exits the cochlea. Despite no threshold shift at 75 dB at 12 kHz, these mice showed decreased wave 1 peak‐to‐peak amplitude with age, suggesting potential dysfunction within the peripheral auditory system, including auditory neurons and hair cells (*p* < 0.001, Figure 1C). However, there was no significant change in wave 1 latency at 12 kHz with age (1 month: 1.858 ± 0.051 ms, 16–18 months: 1.800 ± 0.037 ms, 21–23 months: 1.826 ± 0.029 ms, *p* = 0.138, One‐way ANOVA). Consistent with previous reports at similar ages (Sergeyenko et al. 2013), the reduction in wave 1 amplitude did not correlate with functional loss of OHCs, measured from distortion product otoacoustic emission (DPOAE) thresholds (Figure 1D). Instead, ribbon synapses were lost significantly with age in IHCs (Figure 1F,G). Overall, CBA/CaJ mice present a mild hearing loss with signs of hidden hearing loss.

### 2.2 Apical Hair Cells Become Smaller With Age in CBA/CaJ Mice

To test whether hair cell loss could be responsible for wave 1 reduction, we quantified the number of hair cells with an antibody against myosin 7a (MYO7A), which is used as a hair cell marker (OHC: Figure 1E, IHC: Figure 1F). We found no significant hair cell loss across a 100 μm region at the apical turn of the cochlea before 23 months of age (OHC: *p* = 0.721, Figure 1H, and IHC: *p* = 0.942, Figure 1I), which is consistent with previous studies (Dörje et al. 2024; Sergeyenko et al. 2013). However, we noticed that IHCs from the staining appeared to be much smaller at older ages compared with those at 1 month old (Figure 1F). To quantify the size of hair cells, we performed patch‐clamp recordings to measure the total cell membrane capacitance (*C*_m_), which is proportional to the cell surface area (Solsona et al. 1998). We found that both IHCs and OHCs showed a significant decrease in *C*_m_ already at 9 months compared to 1 month at the apex of the cochlea (9–12 kHz) (IHC: 9–11 months *p* = 0.005, 16–18 months *p* = 0.023, 20–23 months *p* = 0.004, Figure 1H; OHC: 9–11 months *p* < 0.001, 16–18 months *p* < 0.001, Figure 1J). In addition, quantification of the cell volume estimated from the confocal images showed that it was significantly decreased in older IHCs (*p* = 0.009, Figure 1K). Taken together, these data indicate that hair cells become smaller with age. This could potentially be associated with a loss of ion channels in hair cells so as to maintain current density and the function of the cell (Gorur‐Shandilya et al. 2020).

### 2.3 The K^+^ Current in Apical IHCs Becomes Smaller in Aging CBA/CaJ Mice

**FIGURE 2:**
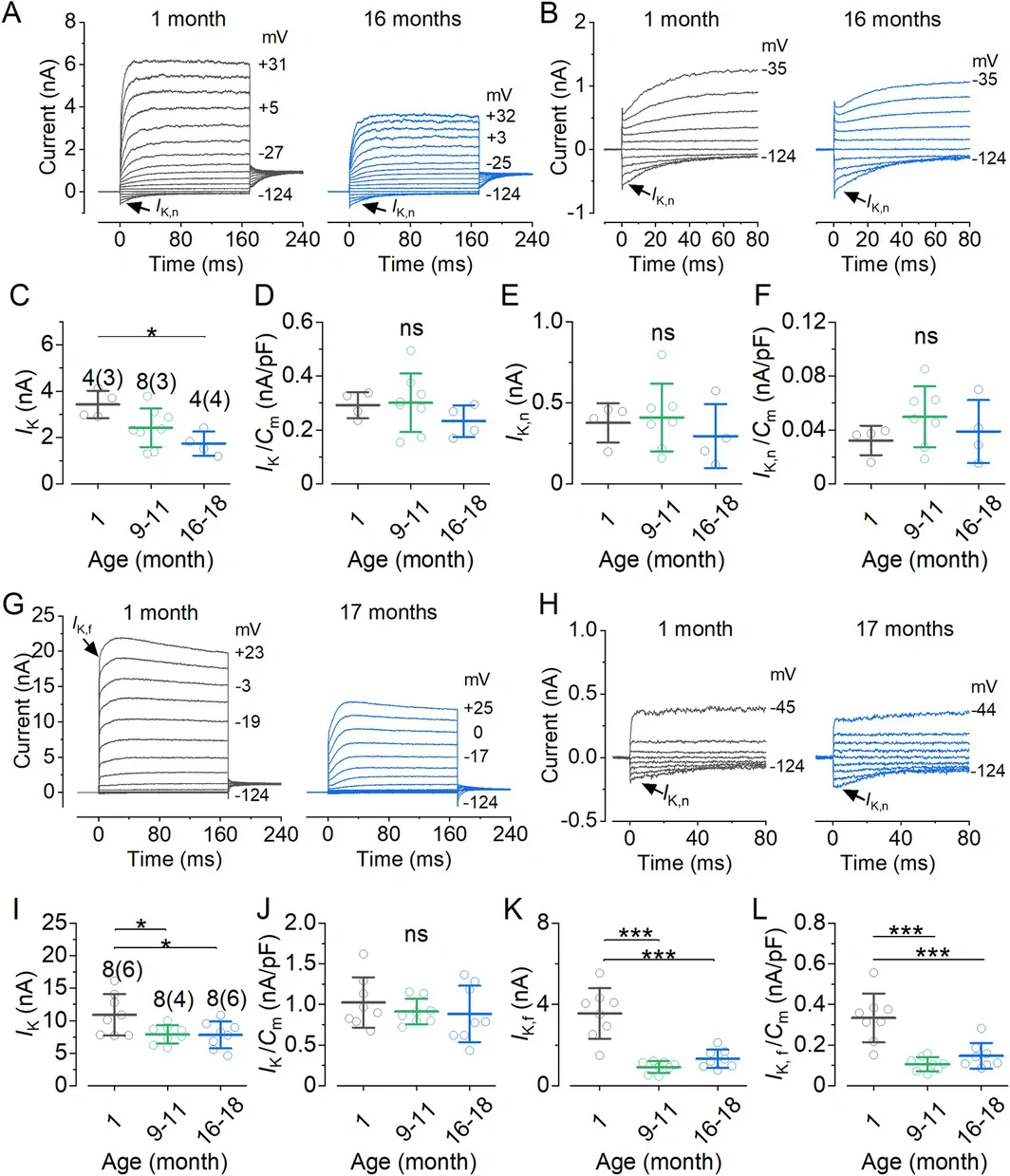
Hair cell current responses decrease with age. Potassium current recorded from aging OHCs (A–F) and IHCs (G–L) in CBA/CaJ mice. Cells were held at −84 mV and currents were recorded using 10 mV depolarising voltage steps from −124 mV. Some of the test potentials are shown by the traces. (A and B) Examples of K^+^ currents recorded from OHCs at 1 and 16 months from a command potentials ranging from −120 to +30 mV (A) and from −120 to −30 mV (B). The presence of *I*_K,n_ is indicated at the beginning of the trace. (C) Size of peak K^+^ current from OHCs measured at a membrane potential of 0 mV. (D) Current density measured by normalizing peak *I*_K_ (C) to the corresponding cell membrane capacitance (*C*_m_). (E) Size of *I*_K,n_ measured at −124 mV. (F) Current density measured by normalizing *I*_K,n_ (E) to the corresponding *C*_m_. (G and H) Examples of K^+^ currents recorded from IHCs at 1 and 17 months. The presence of *I*_K,f_ and *I*_K,n_ is indicated at the beginning of the trace. (I) Size of peak K^+^ current from IHCs measured at a membrane potential of 0 mV. (J) Current density measured by normalizing peak *I*_K_ (I) to the corresponding cell membrane capacitance (*C*_m_). (K) Size of *I*_K,f_ measured at −25 mV. (L) Current density measured by normalizing *I*_K,f_ (K) to the corresponding *C*_m_. Single‐cell recordings are plotted as open symbols. The number of cells (animals) in C–F and I–L is shown above the data points in C and I, respectively. *p < 0.05, ***p < 0.001, one‐way ANOVA followed by Tukey's post hoc test.

To determine whether hair cells lose ion channels with age, we performed whole‐cell patch‐clamp recordings to characterize physiological changes of hair cells from the apical coil of the cochlea (9–12 kHz), as it is technically difficult to isolate and record from the basal coil of the cochlea at these ages. Current responses were elicited using a series of hyperpolarizing and depolarizing voltage steps from a holding potential of −84 mV (Figure 2). For OHCs, we measured the size of the total steady‐state K^+^ current *I*_K_ at 0 mV, and the deactivating *I*_K,n_ (difference between instantaneous and steady‐state inward currents) at 1.5 ms from the current onset at −124 mV (Figure 2A,B). With age, OHCs from CBA/CaJ mice expressed less total current at 16–18 months compared to 1 month (*p* = 0.014, Figure 2C). To investigate whether the reduction in current is related to smaller cell size, we calculated cell current density by normalizing currents to their respective *C*_m_. The resulting current density was not significantly different with age, indicating that the reduction in *I*_K_ is related to cell shrinkage (*p* = 0.452, Figure 2D). In mature mouse OHCs, more than half of *I*_K_ is composed of a negatively‐activating current *I*_K,n_, which is important for maintaining a negative resting membrane potential (Kharkovets et al. 2006; Marcotti and Kros 1999). Compared to *I*_K_, the size of *I*_K,n_ was well‐regulated with age and there was no significant difference in the current density (*p* = 0.629 for *I*_K,n_ and *p* = 0.400 for *I*_K,n_/*C*_m_, Figure 2E,F).

**FIGURE 3:**
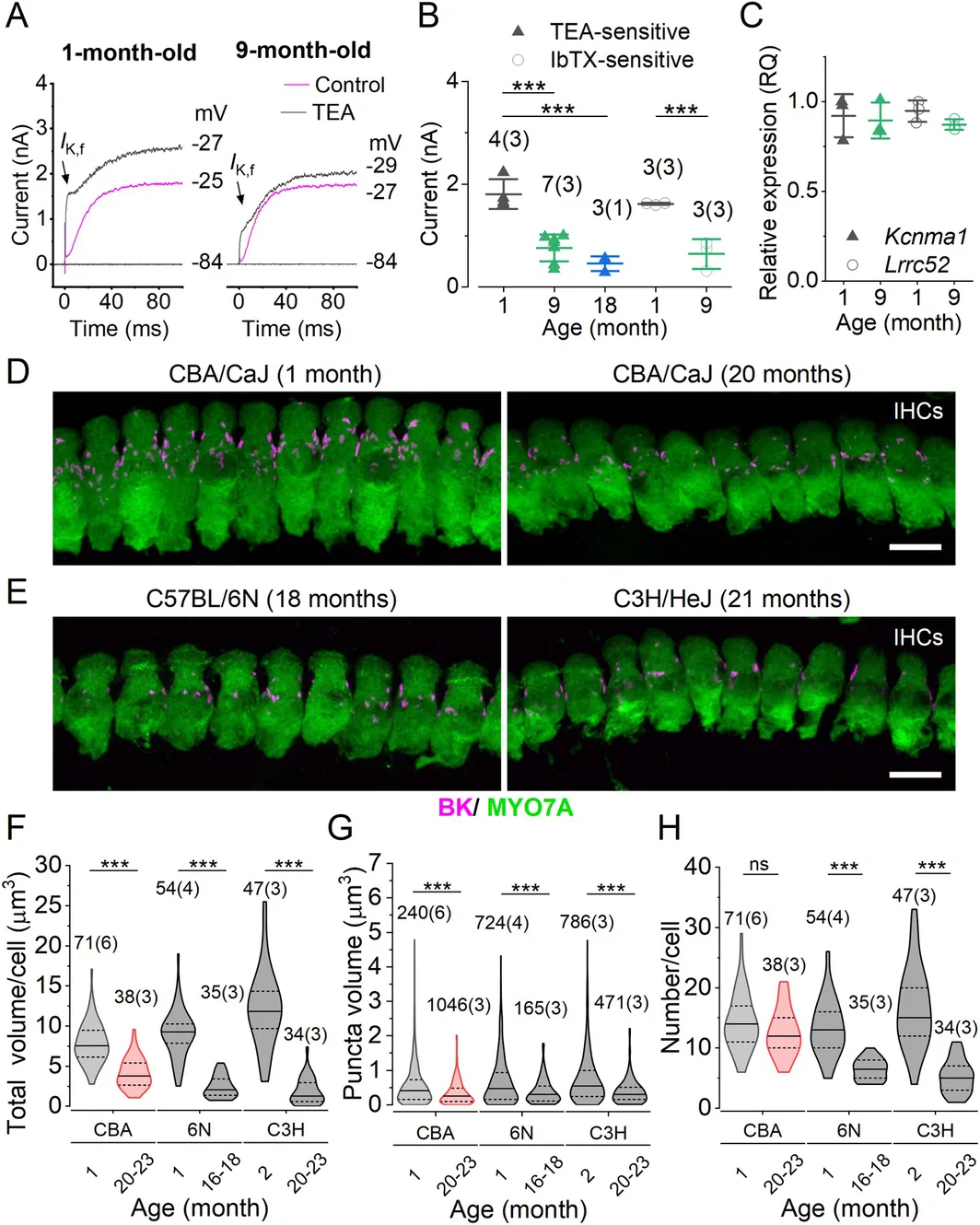
Expression of BK channels was reduced in aging IHCs. (A) Example currents in apical‐coil IHCs from 1‐ and 9‐month‐old CBA/CaJ mice before (control) and after application of 1 mM TEA. Test potentials are shown by the traces. (B) TEA‐ and IbTX‐sensitive current measured at 1.5 ms from the current onset at −25 mV, obtained by current subtraction from recordings shown in A. ***p < 0.001, one‐way ANOVA followed by Tukey's post hoc test. (C) Relative expression of *Kcnma1* and *Lrrc52* from quantitative real‐time PCR of the apical coil from CBA/CaJ mice did not change significantly between 1 and 9 months, one‐way ANOVA. Cochlea from the apical coil of CBA/CaJ (CBA), C3H/HeJ (C3H) and C57BL/6N (6N) mice were labeled with antibodies against a hair cell marker MYO7A (green) and BK channels (red). (D and E) Maximum intensity projections of confocal *z*‐stacks of IHCs from a CBA/CaJ mouse at 1 month and 20 months, a C57BL/6N mouse at 18 months, and a C3H/HeJ mouse at 21 months. (F) Total volume of BK puncta per IHC. Number of cells is shown above the data. (G) Total number of BK puncta per IHC. Number of cells is shown above the data. (H) Volume of individual BK punctum. Number of puncta is shown above the data. Number of animals is shown above the data in brackets in C–E. Scale bar: 10 μm. *p < 0.05; ***p < 0.001; ns, not significant, Kruskal–Wallis followed by Dunn's post hoc test.

For mature mouse IHCs, the total current appears to reduce with age (Figure 2G,H). To quantify this, the size of steady‐state *I*_K_ at 0 mV was measured. The results show that *I*_K_ was significantly reduced with age (*I*_K_ at 1 month vs. 9–11 months *p* = 0.044; vs. 16–18 months *p* = 0.038, Figure 2I). We again calculated cell current density to investigate whether the reduction in current is related to smaller cell size. The results show that, similar to OHCs, the reduction of *I*_K_ in aging IHCs is related to cell shrinkage as there is no significant change in their current density with age (*p* = 0.583 for *I*_K_/*C*_m_, Figure 2J). In mature IHCs, *I*_K_ is composed of *I*_K,s_, *I*_K,n_, and *I*_K,f_. With the same analysis method mentioned above, we found no significant change in *I*_K,n_ with age (1 month: 0.109 ± 0.027 nA, 9–11 months: 0.112 ± 0.051 nA, 16–18 months: 0.100 ± 0.037 nA, *p* = 0.811, one‐way ANOVA). On the other hand, the size of *I*_K,f_ at −25 mV, at 1.5 ms from the current onset, significantly decreased with age (aging vs. 1 month: *p* < 0.001 for *I*_K,f_, Figure 2K). Interestingly, the current density of *I*_K,f_ also significantly decreased with age (*I*_K,f_/*C*_m_ aging vs. 1 month: *p* < 0.001, Figure 2L). To confirm that *I*_K,f_ was carried by the activation of BK channels, we perfused IHCs with two different BK channel blockers, 1 mM tetraethylammonium (TEA) and 100 nM IbTX (Troncoso Brindeiro et al. 2008; Fettiplace and Beurg 2025). As expected, both blockers greatly reduced *I*_K,f_, quantified from the size of TEA‐ and IbTX‐sensitive current (Figure 3A,B, TEA‐sensitive current: 1 vs. 9 month, *p* < 0.001; 1 vs. 18 months, *p* < 0.001, IbTX‐sensitive: *p* < 0.001). Despite reduced *I*_K,f_, mRNA expression of BK channel subunits, *Kcnma1* and *Lrrc52* did not change significantly between 1 and 9 months (Figure 3C, *p* = 0.726). This suggests there were fewer active BK channels in aging IHCs.

Considering the reduction in *I*_K,f_ with age, we further investigated the expression of BK channels that carries *I*_K,f_ in IHCs using immunostaining. The staining was also done in C57BL/6N mice as they were found to express reduced *I*_K,f_ with age previously when compared to C3H/HeJ mice (Jeng, Carlton, et al. 2021). The results show that BK channels were expressed at the neck region of the IHCs from aging CBA/CaJ, C57BL/6N and C3H/HeJ mice (Figure 3D,E). By quantifying all puncta per cell using the Arivis software, we found the total volume of anti‐BK signals for each cell was significantly decreased in aging animals (CBA/CaJ at 1 month vs. 20–23 months: *p* < 0.001; 6 N at 1 month vs. 16–18 months: *p* < 0.001; C3H at 2 months vs. 20–23 months: *p* < 0.001, Figure 3F).

To test whether the decrease of total BK expression is associated with the size and the volume of BK puncta, we analyzed each punctum separately. We found that the average BK volume per single punctum was reduced in all aging mice (CBA/CaJ at 1 month vs. 20–23 months: *p* < 0.001, C3H/HeJ at 1 month vs. 20–23 months: *p* < 0.001, C57BL/6N at 1 month vs. 16–18 months: *p* < 0.001, Figure 3G). However, the number of puncta was significantly lower in aging C3H/HeJ and C57BL/6N mice (CBA/CaJ at 1 month vs. 20–23 months: *p* > 0.999; C3H/HeJ at 1 month vs. 20–23 months: *p* < 0.001, C57BL/6N at 1 month vs. 16–18 months: *p* < 0.001, Figure 3H). This suggests that IHCs in aging CBA/CaJ do not share the same aging process as C3H/HeJ and C57BL/6N mice. In CBA/CaJ IHCs, the decreasing *I*_K,f_ with age is associated with less BK expression per punctum on the membrane.

Overall, these data indicate that although aging hair cells compensate for the decreasing cell size by changing their total K^+^ current profiles, IHCs still fail to maintain their rapid‐activating K^+^ responses.

### 2.4 Aging Apical IHCs Have More Depolarised Voltage Responses

**FIGURE 4:**
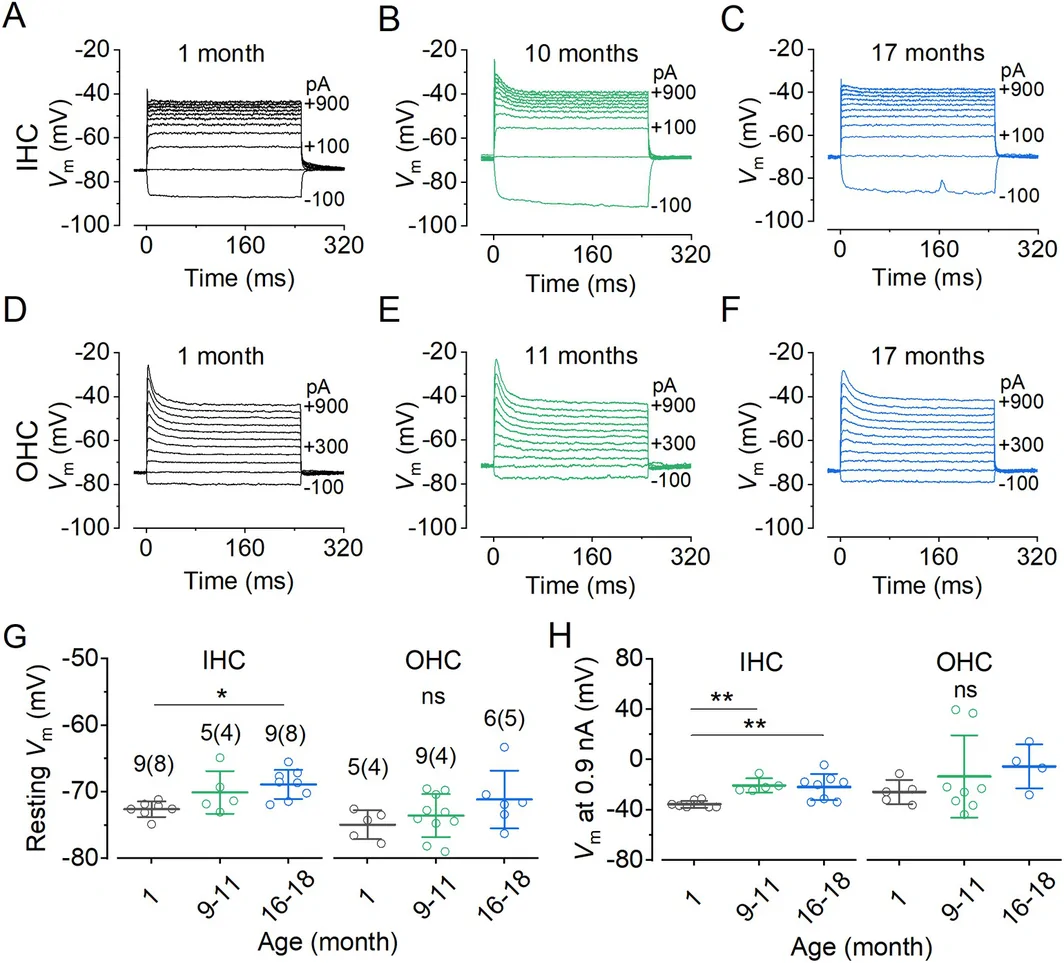
Aging IHCs become more depolarised at rest. (A–F) Examples of voltage responses elicited by applying a series of 0.1 nA current injections to inner hair cells (IHCs) (A–C) and outer hair cells (OHCs) (D–F) from CBA/CaJ mice at 1, 10 and 17 months. (G) Resting membrane potential (*V*_m_) recorded from 1‐, 9–11 and 16–18‐month‐old CBA/CaJ mice. (H) Peak voltage responses in hair cells elicited with 0.9 nA current injection in CBA/CaJ mice at 1, 9–11 and 16–18 months. Single‐cell recordings are plotted as open symbols. The number of cells and animals in G and H is shown above the data points in G. The number of animals is in brackets. **p < 0.01, *p < 0.05, one‐way ANOVA followed by Tukey's post hoc test; ns, not significant.

Since the basolateral membrane currents modulate the receptor potential, we next measured voltage responses in IHCs with age in patch‐clamp recordings. Hyperpolarizing and depolarizing current injections elicit fast and graded voltage responses in both types of hair cells (IHC: Figure 4A–C, OHC: Figure 4D–F). The resting membrane potential (*V*_m_) at 0 current injection in IHCs but not OHCs was significantly more depolarized with age (IHC 1 vs. 16–18 months: *p* = 0.007, OHC: *p* = 0.191, Figure 4G). We further measured the peak voltage responses elicited with 0.9 nA current injection. The current injection triggered a higher membrane potential in aging IHCs but not OHCs (IHC 1 vs. 9–11 months: *p* = 0.008, IHC 1 vs. 16–18 months: *p* = 0.006, OHC: *p* = 0.484, Figure 4H). These data together provide evidence that aging CBA/CaJ IHCs had a more depolarised resting membrane potential ex vivo, potentially due to a decreased overall *I*_K_.

### 2.5 The Mechanoelectrical Transducer Current Is Maintained in Aging Apical IHCs

**FIGURE 5:**
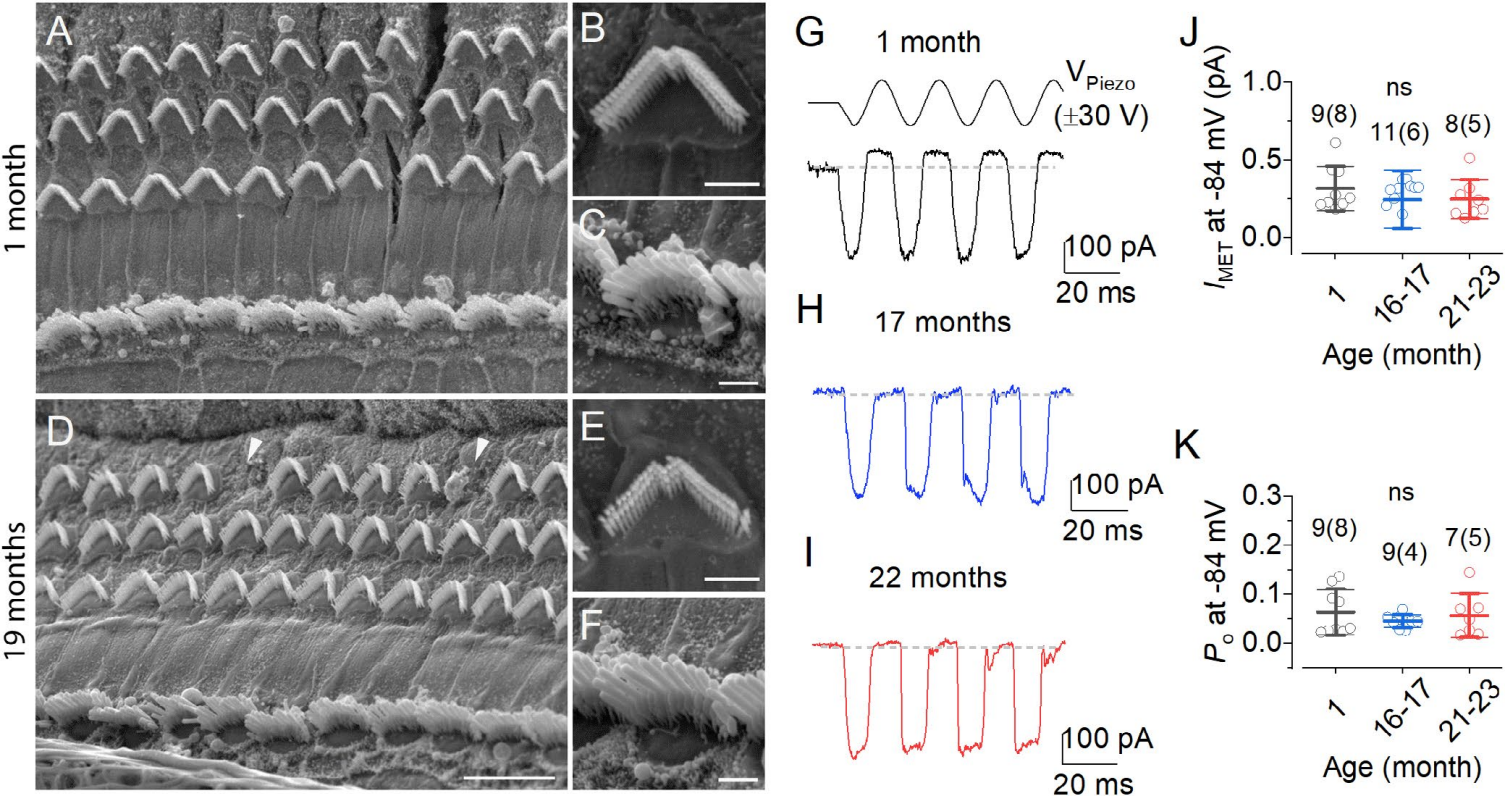
Mechanoelectrical transducer (MET) apparatus in aging hair cells. (A–F) Scanning electron microscopy (SEM) showing the gross morphology of apical‐coil hair cells from CBA/CaJ mice at 1 (A–C) or 19 (D–F) months. Some OHCs were missing at 19 months (arrowheads) but no obvious morphological changes were found in stereociliary bundles from outer hair cells (B and E) and inner hair cells (C and F). (G–I) Examples of saturating MET currents recorded from IHCs of CBA/CaJ mice at 1 (G), 17 (H), and 22 (I) months. MET currents were triggered by applying sinusoidal stimuli of 50 Hz to hair bundles from a holding potential of −84 mV. (J‐K) Maximal size (J) and resting open probability (K) measured from MET current recordings in CBA/CaJ mice with age. Number of cells (animals in brackets) is shown above the data. Single‐cell recordings are plotted as open symbols. ns, not significant, one‐way ANOVA.

We next investigated the MET apparatus in aging CBA/CaJ mice to test whether the decrease in wave 1 amplitude is due to impaired mechanoelectrical transduction. Using scanning electron microscopy, the stereociliary bundles in IHCs and OHCs had uniformly staircase structure without obvious morphological defects (Figure 5A–F). To test if the MET current is unaffected, we performed single‐cell patch‐clamp recordings in aging IHCs from 1‐, 17‐, and 22‐month‐old mice while delivering a sinusoidal bundle displacement stimulus using a fluid‐jet (Figure 5G–I). The maximum size of the MET current at a holding potential of −84 mV was measured from the current difference between the peak current during excitatory bundle displacement and when the MET channels are fully closed during inhibitory stimuli. The results show that the maximum MET current size did not change significantly with age (*p* = 0.549, Figure 5J). The resting open probability (*P*_o_) of the MET current at a holding potential of −84 mV, which is the proportion of MET current active at rest, was also comparable across ages (*p* = 0.591, Figure 5K). These recordings suggest that the MET current in CBA/CaJ mice was not affected during aging, and as such did not contribute to wave 1 reduction with age in these animals.

### 2.6 Efferent Fibers Re‐Innervate Aging IHCs

**FIGURE 6:**
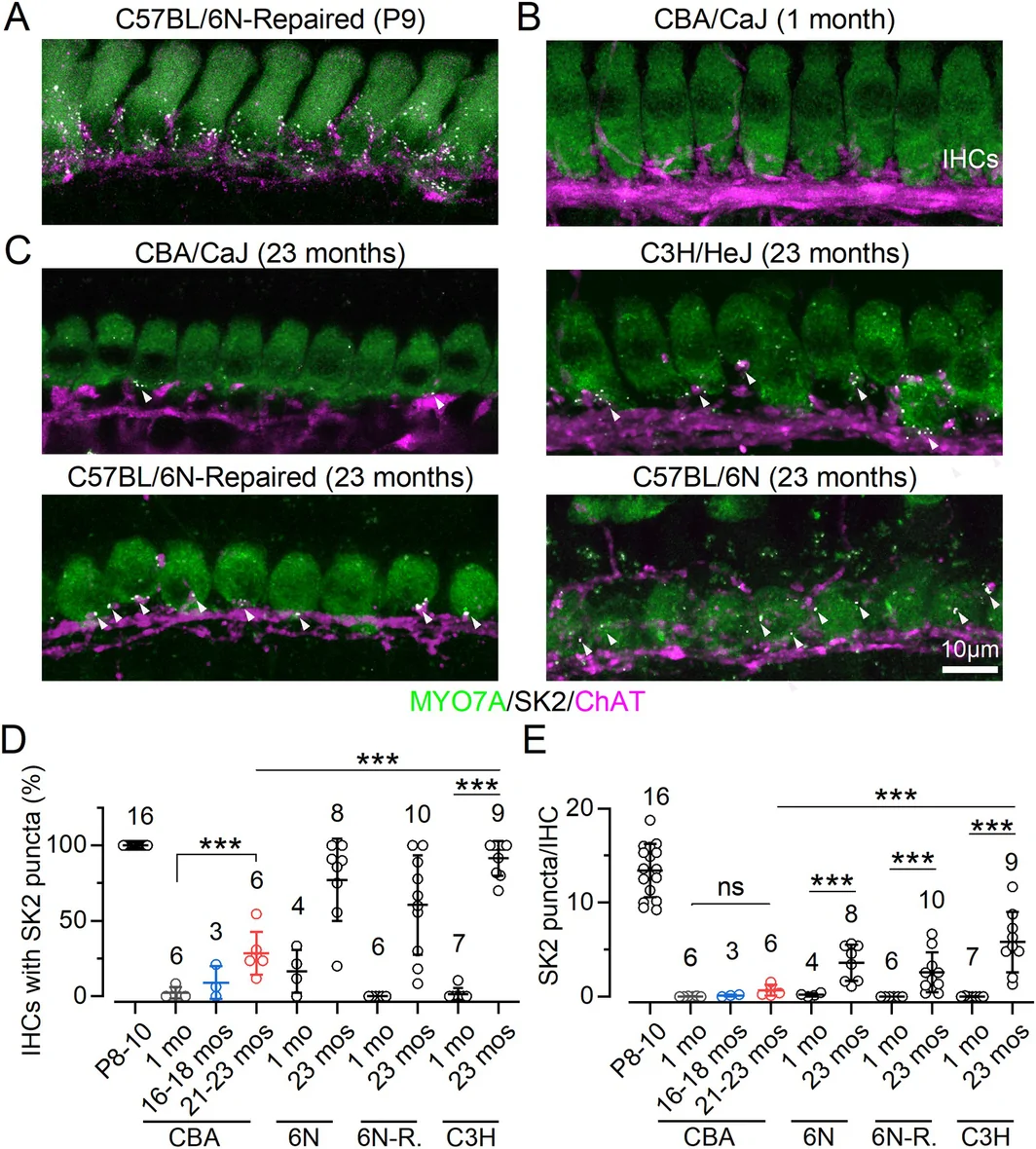
Efferent synapses contacting aging inner hair cells. Cochleae from a 100 μm region of the apical coil from aging CBA/CaJ (CBA), C3H/HeJ (C3H), C57BL/6N (6N), and 6N‐Repaired (6N‐R) mice were labeled with antibodies against a hair cell marker MYO7A (green), SK2 channels (gray), and ChAT (magenta). (A) Maximum intensity projections of confocal *z*‐stacks from apical‐coil IHCs of mice at P9 and 1 month. (B) Maximum intensity projections of confocal *z*‐stacks from apical‐coil IHCs of mice at 23 months. (C) Percentage of IHCs showing SK2 puncta with age. (D) Number of SK2 puncta per IHC with age. Data from 1 month 6N, 6N‐R and C3H are from (Jeng, Ceriani, et al. 2020; Jeng, Johnson, et al. 2020). Data are plotted as mean ± SD. The number of animals is shown above the data. Single counts are plotted as open symbols. ***p < 0.001, one‐way ANOVA followed by Tukey's post‐test; ns, not significant.

During hair cell maturation, the input from cholinergic efferent fibers activates nicotinic acetylcholine receptors (nAChRs) in hair cells. This triggers the opening of small conductance calcium‐activated potassium channels (SK2) that drive cell hyperpolarization (Glowatzki and Fuchs 2000; Figure 6A). After hearing onset, mouse IHCs lose contact with efferent neurons and their ability to respond to acetylcholine (Glowatzki and Fuchs 2000; Marcotti et al. 2004; Figure 6B). With age, IHCs from C57BL/6 mice establish functional connections with inhibitory efferent neurons (Lauer et al. 2012; Zachary and Fuchs 2015; Jeng, Carlton, et al. 2021).

It was proposed previously that reduced MET current from hair cells causes efferent‐fiber re‐innervation of mature IHCs (Corns et al. 2018; O'Connor et al. 2024). If this hypothesis is true, IHCs from CBA/CaJ mice should not be innervated by efferent fibers as they have a near‐normal MET current size. We thus visualized efferent fiber re‐innervation using immunostaining in mice from four aging strains: CBA/CaJ, C3H/HeJ, and 6N‐Repaired (representing late‐onset ARHL models), and C57BL/6N (representing early‐onset ARHL models). We used antibodies against choline acetyltransferase (ChAT) for efferent neurons, and SK2, a small conductance Ca^2+^‐activated K^+^ channel that is present in the post‐synaptic IHCs. We found that the number of IHCs with SK2 puncta significantly increased with age in CBA/CaJ mice (1 month vs. 21–23 months: *p* < 0.001, Figure 6C,D). This number was significantly lower compared to that from C3H/HeJ mice (CBA/CaJ vs. C3H: *p* < 0.001; vs. C57BL/6N: *p* = 0.028, vs. 6N‐Repaired: *p* = 0.076, Figure 6D). CBA/CaJ mice also show fewer SK2 puncta than C3H/HeJ, indicating that the difference between strains is evident among CBA/CaJ and C3H/HeJ mice (*p* < 0.001, Figure 6E). The persistence of efferent re‐innervation in aging IHCs from various ARHL models suggests that this rewiring process is more likely driven by the general aging process rather than resulting from defects in MET currents.

## 3 Discussion

ARHL is the most prevalent sensory disorder, associated with complex genetic and environmental factors. Understanding the onset of ARHL is crucial for developing early treatment interventions that precede irreversible hearing loss. A primary pathology preceding hearing threshold elevation is synaptopathy, though the underlying mechanism is unclear. In this study, we aimed to determine whether hair cells exhibit functional degeneration with age and provide evidence that aging hair cells become smaller and display a decreased K^+^ current profile, which occurs prior to the elevation of hearing thresholds.

Our results highlight that aging hair cell defects involve changes in ion channels, specifically reduced total basolateral currents and BK currents. Although the reduced cell size correlates with the total current response, it is insufficient to account for the significant reduction in BK channel currents (Figure 2L). We found that the volume of anti‐BK channel staining decreased with age, although its expression remained restricted to the neck region of IHCs (Figure 3). This suggests that the protein network at the neck region may be compartmentalized and not simply affected by general cell shrinkage with age. Functionally, since BK currents are crucial for hair cell repolarization, the loss of this current may impair the temporal accuracy of sound transduction (Kurt et al. 2012). This might underlie hidden hearing loss in ARHL, which involves difficulties in understanding speech related to the temporal processing of sounds (Gordon‐Salant et al. 2011; Marmel et al. 2013). Furthermore, the role of BK channels may extend beyond modulating hearing sensitivity. Mutations in the BK channel gene (*Kcma1*) do not cause hearing threshold changes at young ages but increase susceptibility to noise‐induced and ARHL, suggesting a long‐term consequence of BK dysfunction (Rüttiger et al. 2004; Pyott et al. 2007). As BK channels are also expressed in OHCs at basal regions of the cochlea (Wersinger et al. 2010; Rohmann et al. 2015), they may also be involved in high‐frequency vulnerability in ARHL. Therefore, our findings of reduced BK expression in aging mice support the recent hypothesis that ion channels, such as the BK channel, might be a potential therapeutic target for ARHL (Peixoto Pinheiro et al. 2021). However, further investigation is required to distinguish the impact of reduced auditory hair cell ion channels from other aging pathways.

Despite changes in the basolateral profiles, CBA/CaJ mice did not show a decrease in MET current up to about 21–23 months of age (Figure 4). However, as the total basolateral current responses were smaller with age (Figure 2), the current balance between MET current and basolateral current may not be maintained for a normal receptor potential. This is supported by the observation of a more depolarized resting membrane potential in older (16–18 months) compared with young (1 month) IHCs (Figure 5G). Considering that in vivo hair cells rest at a higher membrane potential and have a larger MET current response, changes observed ex vivo might have a greater physiological impact in vivo (Johnson 2015). This might reflect a change in the operational range of IHCs and thus affect the spontaneous synaptic vesicle release rate at rest for IHCs (Tobón and Moser 2024).

In this study, we found that IHCs in CBA/CaJ mice express SK2 channels localized near efferent fibers. While this organization was previously identified as likely involving the lateral olivocochlear (LOC) fibers (Lauer et al. 2012; Zachary and Fuchs 2015; Jeng, Carlton, et al. 2021), the full impact of re‐innervation is still unclear. If efferent neurons modulate aging IHCs similarly to immature hair cells, the re‐innervation might influence the organization of synapses as reported during developmental stages (Johnson et al. 2013; Clause et al. 2017). Another possible hypothesis is that efferent neurons simply respond to physiological changes in IHCs. Nevertheless, it is unlikely that transduction via hair bundles orchestrates the re‐innervation, as the MET current did not change with age in CBA/CaJ and 6N‐Repaired mice despite an average of 30% and 60% of aging IHCs being contacted by efferent fibers, respectively (Figure 6D, Jeng, Carlton, et al. 2021).

Overall, the auditory system's aging process differs significantly between mouse strains regardless of their progression of hearing loss. For example, there is a significant difference in the number of re‐innervated efferent contacts between CBA/CaJ and C3H/HeJ mice (Figure 6). In aging CBA/CaJ mice, hair cells express reduced *I*_K_ and *I*_K,f_ (Figure 2), a feature observed in C57BL/6N but absent in C3H/HeJ mice (Jeng, Johnson, et al. 2020; Jeng, Carlton, et al. 2021). Consistent with these changes, previous literature indicates that CBA but not C3H mice begin to lose synaptic ribbons at a similar age period (Parthasarathy and Kujawa 2018; Jeng, Carlton, et al. 2021; Dörje et al. 2024). This divergence between CBA and C3H mice is intriguing because, despite originating from the same initial cross, aging CBA mice exhibit physiological changes distinct from those in C3H mice. These differences underscore the significant influence of genetic background on auditory aging.

To summarize, aging apical hair cells in CBA/CaJ mice exhibit signs of progression towards immature cells, including smaller cell size, reduced expression of BK channels, and re‐innervation by efferent neurons. However, due to technical difficulties, it remains unknown whether these changes occur in basal hair cells and cause hearing loss in later life. In conclusion, our findings demonstrate that the hair cells of aging CBA/CaJ mice undergo progressive functional degeneration that precedes the onset of measurable hearing loss, suggesting cellular deficits as a key step in auditory aging pathology.

## 4 Materials and Methods

### 4.1 Tissue Preparation for Ex Vivo Recordings

Patch clamp recordings were obtained from hair cells within the 9–12 kHz region of the cochlear apical coil. Cochleae were dissected out in an extracellular solution composed of (in mM): 135 NaCl, 5.8 KCl, 1.3 CaCl_2_, 0.9 MgCl_2_, 0.7 NaH_2_PO_4_, 5.6 D‐glucose, 10 HEPES‐NaOH. Sodium pyruvate (2 mM), amino acids and vitamins were added from concentrates (Thermo Fisher Scientific, UK). The pH was adjusted to 7.48 with 1 M NaOH, and the final osmolality of the solution was measured (~306 mmol/kg). The dissected apical coil was then transferred to a microscope chamber and immobilized via a nylon mesh attached to a stainless‐steel ring. The chamber (volume 2 mL) was perfused using a peristaltic pump (Cole‐Palmer, UK) and mounted on the stage of an upright microscope (Olympus BX51, Japan) with Nomarski Differential Interference Contrast (DIC) optics. The individual stereocilia rows of the hair cells were identified using a 60× water immersion objective and a 15× eyepiece.

### 4.2 Whole‐Cell Electrophysiology

Patch clamp recordings were performed at room temperature (20°C–24°C) using an Optopatch amplifier (Cairn Research Ltd., UK). Patch pipettes were pulled from soda glass capillaries (1413027; Hilgenberg) and had a typical resistance in extracellular solution of 2–3 MΩ. The patch pipette intracellular solution contained (in mM): 131 KCl, 3 MgCl_2_, 1 EGTA‐KOH, 5 Na_2_ATP, 5 HEPES‐KOH, 10 Na‐phosphocreatine (pH was adjusted with 1 M KOH to 7.28, ~294 mmol/kg). Data acquisition was controlled by pClamp software using Digidata 1440A and 1550 (Molecular Devices, USA). To reduce the electrode capacitance, patch electrodes were coated with surf wax (Mr. Zoggs SexWax, USA). Recordings were low‐pass filtered at 2.5 kHz (8‐pole Bessel), sampled at 5 kHz and stored on a computer for off‐line analysis (Clampfit, Molecular Devices and Origin 2020, OriginLab). For voltage‐clamp experiments, membrane potentials were corrected off‐line for the residual series resistance after compensation (60%–80%) and the liquid junction potential (LJP) of −4 mV, which was measured between electrode and bath solutions. Holding currents were plotted as zero current to allow a better comparison between recordings. Voltage clamp protocols are set to a holding potential of −84 mV or −64 mV depending on the protocol used.

For mechanoelectrical transducer (MET) current recordings, the hair bundles of hair cells were displaced using a fluid jet from a pipette driven by a 25 mm diameter piezoelectric disc (Corns and Marcotti 2016; Jeng, Harasztosi, et al. 2021). The pipette was pulled from borosilicate glass (1423027; Hilgenberg) to a final overall length of 5.3–5.5 cm. The fluid jet pipette tip had a diameter of 8–10 μm and was positioned near the hair bundles to elicit a maximal MET current. Mechanical stimuli were applied as 50 Hz sinusoids (filtered at 1 kHz, 8‐pole Bessel). Prior to positioning of the fluid jet near the hair bundles, any steady‐state pressure was removed by monitoring the movement of debris in front of the pipette. The use of the fluid‐jet allows for the efficient displacement of the hair bundles in both the excitatory and inhibitory directions, which is essential for reliable measurements of the resting open probability of the MET channels.

### 4.3 Auditory Brainstem Responses

Following the onset of anesthesia (see Ethics statement below) and the loss of the retraction reflex with a toe pinch, mice were placed in a soundproof chamber (MAC‐3 acoustic chamber, IAC Acoustics, UK). Male and female mice were placed on a heated mat (37°C) with the animal's pinna being positioned at a distance of 10 cm from the loudspeaker. Two subdermal electrodes were placed under the skin behind the pinna of each ear (reference and ground electrode), and one electrode halfway between the two pinnae on the vertex of the cranium (active electrode) as previously described (Ingham et al. 2011). Sound stimuli were delivered to the mouse ear by a loudspeaker (MF1‐S, Multi Field Speaker; Tucker‐Davis Technologies, USA), which was calibrated with a low‐noise microphone probe system (ER10B+, Etymotic, USA). Experiments were performed using customized software (Ingham et al. 2011) driving an RZ6 auditory processor (Tucker‐Davis Technologies). ABR thresholds, for which stimuli were delivered as white noise clicks and pure tone stimuli of frequencies at 6, 12, 18, 24, 30, 36, and 42 kHz, were defined as the lowest sound level where any recognizable feature of the waveform was visible. Stimulus sound pressure levels of up to 95 dB SPL were presented in steps of 5 dB SPL (averaged over 256 repetitions). Tone bursts were 5 ms in duration with a 1 ms on/off ramp time presented at a rate of 42.6/s.

### 4.4 Distortion Product Otoacoustic Emissions (DPOAEs)

Recordings were performed in a soundproof chamber (MAC‐3 Acoustic Chamber, IAC Acoustics, UK). DPOAEs were recorded at 2f_1_‐f_2_ in response to primary tones f_1_ and f_2_, where f_2_/f_1_ = 1.2 and f_2_ = 6.5–39.2 kHz. The f_1_ level (L_1_) was set from 20 to 80 dB in 10 dB increments, and the f_2_ level (L_2_) was set equal to L_1_. Frequency pairs of tones were presented directly into the ear canal by means of a metal coupler connected to two calibrated loudspeakers (MF1‐S, Multi Field Speaker; Tucker‐Davis Technologies, USA). DPOAEs were recorded by a low‐noise microphone (ER10B+: Etymotic Research Inc., USA). The response signal was averaged over 500 repetitions. Measurements were performed using BioSigRZ software driving an RZ6 auditory processor (Tucker‐Davis Technologies). DPOAE thresholds were defined as the minimal sound level where the DPOAEs were above two standard deviations of the noise. Thresholds were plotted against the geometric mean frequency of f_1_ and f_2_.

### 4.5 Scanning Electron Microscopy (SEM)

The dissected mouse cochleae were initially fixed by a very gentle intra‐labyrinthine perfusion of fixative using a pipette tip through the round window. Following perfusion, the cochleae were immersed in the fixative for 2 h at room temperature. The fixative contained 2.5% glutaraldehyde in 0.1 M sodium cacodylate buffer and 2 mM CaCl_2_ (pH 7.4). After fixation, the organ of Corti was exposed by removing the bone from the apical coil of the cochleae and then post‐fixed in 1% osmium tetroxide in the cacodylate buffer for 1 h. For osmium impregnation, cochleae were incubated in solutions of saturated aqueous thiocarbohydrazide (20 min) alternating with 1% osmium tetroxide in cacodylate buffer (2 h) twice (OTOTO technique). The cochleae were then dehydrated through an ethanol series (70%–100%) and critical‐point dried using CO_2_ as the transitional fluid (Leica EM CPD300) and mounted on specimen stubs using conductive silver paint (Agar Scientific, Stansted, UK). The apical coil of the organ of Corti was examined at 10 kV using a Tescan Vega3 LMU scanning electron microscope at the School of Biosciences Electron Microscopy Facility at the University of Sheffield.

### 4.6 Immunofluorescence Microscopy

This work was performed by fixing the inner ear with 4% paraformaldehyde in phosphate‐buffered saline (PBS, pH 7.4) for 20 min at room temperature. Cochleae were washed three times in PBS for 10 min and finely dissected for the Organ of Corti. Following dissection, samples were incubated in PBS supplemented with 5% normal goat or horse serum and 0.5% Triton X‐100 for 1 h at room temperature. The samples were immunolabeled with primary antibodies overnight at 37°C, washed three times with PBS and incubated with the secondary antibodies for 1 h at 37°C. Antibodies were prepared in 1% serum and 0.5% Triton X‐100 in PBS. Primary antibodies were: mouse anti‐myosin 7a (1:1000, Developmental Studies Hybridoma Bank, #138‐1C), rabbit anti‐myosin 7a (1:200, Proteus Biosciences, #25‐6790), rabbit anti‐SK2 (1:500, Sigma‐Aldrich, P0483), goat anti‐choline acetyltransferase (ChAT, 1:500, Millipore, AB144P), and mouse‐IgG1 anti‐BK channel (1:200; NeuroMab, 75‐408). Secondary antibodies were species appropriate Alexa Fluor conjugated antibodies. Samples were mounted in VECTASHIELD (H‐1000). The images from the apical cochlear region (8–12 kHz) were captured with a Zeiss LSM 880 Airyscan equipped with a Plan‐Apochromat 63× Oil DIC M27 objective. The microscope is part of the Wolfson Light Microscope Facility at the University of Sheffield. Image stacks were processed with Fiji ImageJ software, and volume analyses were processed with Zeiss Arivis software.

### 4.7 Quantitative Real‐Time PCR (qRT‐PCR)

Apical coils of cochleae were dissected from mice at 1 and 9 months of age and frozen in liquid nitrogen before processing. RNA was isolated using the RNeasy Micro Kit (74004; QIAGEN) and RNA quantity and quality were verified with the NanoDrop. Equal amounts of cDNA were synthesized using the High‐Capacity RNA‐to‐cDNA Kit (4387406; Applied Biosystems). Quantitative real‐time PCRs were performed using a QuantStudio 12 K Flex Real‐Time PCR System and PowerUp SYBR Green Master Mix (A25780; Applied Biosystems). Samples were run in triplicate on a 384‐well plate (E1042‐3840; StarLab), with inclusion of appropriate no‐template controls. Primers used were: *Kcnma1* (5′‐GCCAGAGTCAAGATAGAGTCAGC‐3′ and 5′‐GATGATCCTGATCTTTGGGTGG‐3′); *Lrrc52* (5′‐CTGTACCTAAGTGGGAACCC‐3′ and 5′‐AGAAGATGTAGTCCTGCTGGT‐3′). Expression levels were normalized using the housekeeping genes *Hprt1* (5′‐CCAGCGTCGTGATTAGCGATG‐3′ and 5′‐GCCACAATGTGATGGCCTCC‐3′) and *Tbp* (5′‐GGAGCCAAGAGTGAAGAACAATC‐3′ and 5′‐TCAGCACAAGGCCTTCCAGC‐3′), analyzed using the $2^{-\Delta \Delta C_{T}}$ method.

### 4.8 Statistical Analysis

Statistical comparisons of means were made using Student's two‐tailed *t*‐test or, for multiple comparisons, analysis of variance (one‐way and two‐way ANOVA followed by Tukey's or Sidak's post hoc test). Only mean values with a similar variance between groups were compared and values are quoted in the text and figures as means ± standard deviation. In the presence of an upper detection limit of equipment, such as when determining hearing thresholds, nonparametric Kruskal–Wallis ANOVA was used and values are quoted in text and figures as median ± median absolute deviation. *p* < 0.05 was selected as the criterion for statistical significance.

## Author Contributions

All authors helped with the analysis of the data. J.‐Y.J. conceived and coordinated the study. All authors approved the final version of the manuscript. All authors agree to be accountable for all aspects of the work in ensuring that questions related to the accuracy or integrity of any part of the work are appropriately investigated and resolved.

## Funding

This work was supported by the BBSRC (BB/X000567/1) and the RNID (G106) to S.L.J.; the Wellcome Trust (300350/Z/23/Z) to A.J.C.; the BBSRC (BB/Z514743/1) to J.‐Y.J.

## Disclosure

Sustainability Statement: To promote environmental sustainability, the Hearing Research group at the University of Sheffield utilizes resource‐efficient laboratory workflows and holds a Gold LEAF accreditation. All research activities are conducted with a commitment to minimizing environmental impact. The laboratory adheres to sustainable practices, including the optimization of ultra‐low temperature storage, the reduction of single‐use plastics alongside the recycling of non‐contaminated items, and the routine shutdown of equipment when not in use to reduce energy consumption. Material waste is minimized through the consolidation of reagent ordering to reduce shipping emissions, as well as the responsible segregation and recycling of non‐hazardous lab plastics.

## Ethics Statement

The animal work was licensed by the UK Home Office under the Animals (Scientific Procedures) Act 1986 (PPL_PCC8E5E93) and was approved by the University of Sheffield Ethical Review Committee (180626_Mar). Both males and females were used for this study. For ex vivo experiments, mice were killed by cervical dislocation followed by decapitation. For in vivo auditory brainstem responses (ABRs) and distortion product otoacoustic emissions (DPOAEs), mice were anesthetized using an intraperitoneal injection of ketamine (100 mg/kg body weight, Fort Dodge Animal Health, USA) and xylazine (10 mg/Kg, Rompun, Bayer HealthCare LLC, USA). At the end of the ABR recordings, mice were either culled by cervical dislocation or recovered from anesthesia with an intraperitoneal injection of atipamezole (1 mg/Kg, Antisedan, Orion Corporation, Finland).

## Conflicts of Interest

The authors declare no conflicts of interest.

## Data Availability

The data that support the findings of this study are available from the corresponding author upon reasonable request.
